# Adaptation, validity and reliability of the modified painDETECT questionnaire for patients with subacromial pain syndrome

**DOI:** 10.1371/journal.pone.0211880

**Published:** 2019-02-06

**Authors:** Barbara C. Boer, Jolanda Boetje, Martin Stevens, Inge van den Akker-Scheek, Jos J. A. M. van Raay

**Affiliations:** 1 Department of Orthopedics, Martini Hospital Groningen, Groningen, The Netherlands; 2 Department of Orthopedics, University of Groningen, University Medical Center Groningen, Groningen, The Netherlands; Florida State University, UNITED STATES

## Abstract

**Background:**

The identification of a neuropathic component to subacromial pain may lead to different pain management strategies. The purpose of this study was to adapt the Dutch modified painDETECT (mPDQ-NL) Knee, which discriminates between nociceptive and neuropathic pain, to fit patients with subacromial pain syndrome and subsequently assess its validity and reliability.

**Methods:**

The mPDQ-NL Knee was adapted into the mPDQ-NL Shoulder to fit and use for patients with subacromial pain syndrome. The study population consisted of patients suffering from subacromial pain syndrome who were asked to fill in the Dutch mPDQ-NL Shoulder, the self-completed Leeds Assessment of Neuropathic Symptoms and Signs (S-LANSS) and the Disabilities of the Arm, Shoulder and Hand (DASH) to determine construct validity (structural validity, hypotheses testing) of the mPDQ-NL Shoulder. Regarding reliability, internal consistency was determined and the mPDQ-NL Shoulder was assessed a second time with a two-week interval to determine measurement error and reliability.

**Results:**

A total of 107 patients were included in the validity analysis and 58 in the reliability analysis. Validity (86% of predefined hypotheses met), internal consistency (Cronbach’s alpha 0.8) and reliability (ICC 0.7) of the mPDQ-NL Shoulder were good, however, a systematic bias might be present.

**Conclusion:**

The mPDQ-NL Shoulder was successfully modified from the mPDQ-NL Knee. This study shows that the mPDQ-NL Shoulder seems to reflect neuropathic-like pain symptoms experienced by patients with SAPS. Whether it may also be used as a tool to record change over time or after treatment has to be further assessed.

## Introduction

Subacromial pain syndrome (SAPS) is a common cause of shoulder pain, with a high societal burden [1,2]. There are many contributing pathologies, including subacromial bursitis, rotator cuff tendinopathy, partial thickness rotator cuff tears and concomitant degenerative changes in the acromioclavicular joint [1,3]. It should be preferably treated nonoperatively, although up to half of patients will continue to have pain after several years or have underwent surgery [4,5]. According to the guidelines of the Dutch Orthopaedic Association, surgery can be considered after a year of conservative treatment without clinical improvement [6]. No advantage of surgical treatment over conservative treatment has been shown [7,8].

There is growing evidence that pain in SAPS might not be purely nociceptive, but that central sensitization can be present [9–12]. This augmented pain transmission is characterized by local and generalized lowered pain thresholds and an exaggerated pain response to painful and non-painful stimulation [9,11]. These manifestations may be described as neuropathic pain.

Central hypersensitivity might be associated with large numbers of patients with SAPS who have persistent pain or a limited range of motion for several years despite treatment [4,8]. It is essential to identify patients with SAPS with a neuropathic pain profile, as they could benefit from additional or so far largely unexplored treatment options other than conventional nociceptive pain medication or surgery, and may receive different education strategies. In the setting of future research, identifying subgroups of patients with SAPS with (or without) neuropathic features could help assess the true effectiveness of these new and already existing treatment options. The first step toward individualized treatment, however, is a valid and reliable questionnaire to identify patients with neuropathic pain symptoms in SAPS.

Among existing measures, the painDETECT questionnaire appears the most appropriate for use in SAPS to identify patients with a neuropathic pain profile; it was developed to discriminate between nociceptive and neuropathic pain in adults with chronic low back pain and has proven to be a valid and reliable screening tool [13]. It was modified by Hochman et al. to fit patients suffering from knee osteoarthritis [14]. Rienstra et al. translated and cross-culturally adapted the questionnaire for Dutch knee osteoarthritis patients (mPDQ-NL Knee) [15]. To date, no Dutch questionnaire is available to discriminate between nociceptive and neuropathic pain for patients with SAPS. Hence the objectives of this study were to modify the Dutch mPDQ-NL Knee for use in patients with SAPS (mPDQ-NL Shoulder) and subsequently assess its validity and reliability.

## Methods

The study was approved by the Medical Ethical Committee of University Medical Center Groningen (no. METc 2015–025). The methods, particularly the statistical analyses, have been previously described by Rienstra et al. [15].

### Participants

Patients suffering from SAPS who were receiving any form of conservative treatment or were on the waiting list to undergo surgery were eligible to participate. A random sample of 309 patients with SAPS at the outpatient clinic between April 2015 and October 2016 was obtained from the records of the Department of Orthopedic Surgery of Martini Hospital Groningen, The Netherlands. All patients were diagnosed with SAPS with rotator cuff tendinopathy or chronic subacromial bursitis as contributing pathologies through anamnesis and physical and radiological examination by their orthopedic surgeon. All patients had powerful rotator cuff tests during physical examination. Exclusion criteria were age under 18, other shoulder pathologies (glenohumeral or acromioclavicular osteoarthritis and rotator cuff rupture were ruled out by X-ray and ultrasound in order to provide a homogenous group), previous shoulder surgery, other chronic pain disorders, severe comorbidity, cognitive or psychiatric disorders, and inability to understand written Dutch.

### Procedure

Eligible patients were mailed a letter regarding informed consent, the voluntary nature of the study and the anonymous data-processing methods, together with the mPDQ-NL Shoulder, a questionnaire on demographic characteristics and comorbidities, and a prepaid reply envelope. To assess validity patients also received the self-completed Leeds Assessment of Neuropathic Symptoms and Signs (S-LANSS) [16,17], the Disabilities of the Arm, Shoulder and Hand (DASH) [18,19], and a Visual Analog Scale (VAS) [20]. Patients were asked to return the completed set of questionnaires and were informed that this was considered as informed consent to participate in the study. Patients with bilateral SAPS were asked to complete the questionnaire for the shoulder joint that was most symptomatic. To assess reliability and measurement error, a second mPDQ-NL Shoulder was sent to participants after a two-week interval. In case of incomplete questionnaires or non-response, patients were contacted in order to complete missing items or ask for their participation.

### Measures and questionnaires

#### mPDQ-NL Shoulder

We developed the mPDQ-NL Shoulder from the Dutch mPDQ-NL Knee by replacing the target joint “knee” with “shoulder” in every question, with a few other minor changes related to the anatomical site (i.e. the location on the body map). The pre-final version of the mPDQ-NL Shoulder was tested in a pilot study consisting of 20 patients with SAPS visiting the orthopedic outpatient clinic of Martini Hospital to test for comprehensibility of the questionnaire. No problems or comments were reported while filling out the questionnaire, measured via a response form regarding instructions, unclear questions and any additional comments. See S1 and S2 Appendices for the final mPDQ-NL Shoulder with the scoring system.

The mPDQ-NL Shoulder is a self-report questionnaire consisting of 12 items in four components about neuropathic pain symptoms of the left or right shoulder experienced during the last week. The first component evaluates pain radiation using a body map. The second component assesses pain pattern and course. In the third component pain quality is evaluated for seven items on a six-point Likert scale (burning sensation, tingling or prickling sensation, pain at light touch, sudden pain attacks, pain at cold or warm stimulus, numbness and pain at light pressure). A fourth VAS component evaluates pain intensity on three items (pain at this moment, worst pain in the past week, average pain in the past week). The total score is the sum of the first three components and ranges from -1 to 38 points, with higher scores indicating a more neuropathic pain profile. Cut-off points were chosen in accordance with the original PDQ [13]. A score ≤12 indicates unlikely presence of a neuropathic pain component, a score ≥19 indicates likely presence. A score between 13 and 18 suggests a possible neuropathic pain profile.

#### S-LANSS

The Self-Completed Leeds Assessment of Neuropathic Symptoms and Signs (S-LANSS) is an English-validated and reliable self-report questionnaire to identify pain of predominantly neuropathic origin in patients with chronic pain from any cause [16,17]. It consists of seven items and uses a weighted binary scoring system. The first five items address neuropathic pain symptoms, the last two are related to clinical signs where gently rubbing and pressing the painful area is compared with a non-painful area. The total score ranges from 0 to 24 points. A score of ≥12 suggests pain of predominantly neuropathic origin. The S-LANSS has not been specifically validated for patients with SAPS [17,21,22]. The Dutch version of the S-LANSS used in the present study was translated and cross-culturally adapted according to international guidelines [23].

#### DASH outcome measure

The Disabilities of the Arm, Shoulder and Hand (DASH) is a 30-item valid and reliable self-report questionnaire that measures physical function and symptoms of the past week on a 5-point Likert scale in people with any of several musculoskeletal disorders of the upper limb [18,19]. The total score ranges from 0 to 100 points and a higher score indicates greater disability.

**VAS pain.** Visual Analogue Scales (VAS) are widely used to measure pain. Patients place a marking on a horizontal line that represents their pain, where the left end of the line represents “no pain at all” and the right end “worst pain imaginable”. The found value was rounded up and represents the pain score. Patients were asked to record average shoulder pain at rest and during physical activity for the last week. VAS scales have been reported as valid and reliable measures for pain intensity [20].

### Statistical analyses

Based on Cosmin guidelines a sample size of at least 100 is considered excellent for studies on measurement properties of questionnaires; a sample size of 50 is considered adequate for determining test-retest reliability [24]. Hence we planned a sample size of at least 100 participants to assess the construct validity of the mPDQ-NL Shoulder, and a sample size of at least 50 to establish the test-retest reliability. Patients were subsequently asked to participate.

Statistical analyses were conducted using IBM SPSS, version 22.0 (SPSS Inc., Chicago). A p-value <0.05 was considered to indicate statistical significance. When missing items were present on the mPDQ-NL Shoulder, total score could not be determined. Missing items of the DASH and S-LANSS were treated according to the guidelines proposed by the developers of the used questionnaires. If patients reported no pain on all three VAS items of the fourth component of the mPDQ-NL Shoulder, they were excluded from the analyses. Patient characteristics were reported using descriptive statistics.

Kruskall Wallis tests were used to assess differences between subgroups based on the mPDQ-NL Shoulder scores (≤ 12 points, 13–18 points and ≥ 19 points). Posthoc analyses were performed with Mann-Whitney U tests to determine between groups differences. Correction for multiple comparisons was done by a Bonferroni correction and resulted in a significance level of 0.05/18 tests = 0.003.

#### Construct validity

Validity of the Dutch m-PDQs was expressed in terms of construct validity due to absence of a gold standard. Construct validity is used to determine how well a test measures what it is supposed to measure [25].

**Structural validity.** Structural validity refers to the degree to which items of a questionnaire measure the dimensionality of the construct. While the original PDQ measured pain in patients with chronic low back pain, it had to be investigated whether the items of the mPDQ-NL Shoulder reflect the neuropathic-like symptoms of patients with SAPS. The original PDQ measured two determinative components [13]. Exploratory factor analysis, using Kaiser’s criterion with varimax rotation, was conducted to determine whether the items on the questionnaire form one single overall factor or several [25]. Factors with eigenvalues ≥1.00 were selected; an eigenvalue ≥1 explains more variance than a single observed variable [26].

**Hypotheses testing.** The mPDQ-NL Shoulder was compared with the S-LANSS, DASH, VAS at rest and VAS during physical activity (see Table 1 for predefined hypotheses). The constructs of the mPDQ-NL Shoulder and the S-LANSS are considered the most similar as they both measure neuropathic pain components consequently we expected a strong correlation between both questionnaires. Smaller correlations were expected between the mPDQ-NL Shoulder and DASH, since the latter focuses mainly on disabilities during activities and not specifically on neuropathic pain. As most patients have more pain during exercise, mainly during overhead work which causes impingement, a higher correlation was expected between the mPDQ-NL Shoulder and VAS pain during activities than with VAS pain at rest.

**Table 1:** A priori hypotheses and found correlations between the mPDQ-NL Shoulder and the S-LANSS, DASH, VAS at rest and VAS during physical exercise.

	Expected correlation	Found correlation	Hypotheses confirmed
**S-LANSS**	0.70–0.89	0.55	No
	Higher than VAS physical activity		Yes
	Higher than DASH		Yes
**DASH**	0.26–0.49	0.46	Yes
	Lower than VAS physical activity		Yes
**VAS at rest**	0.26–0.49	0.36	Yes
**VAS physical activity**	0.50–0.69	0.50	Yes

Predefined hypotheses regarding the correlations between the questionnaires were tested using Pearson or Spearman correlation coefficients depending on normality of the distribution of the different scales. They were interpreted according to the criteria set by Domholdt et al., where 0.00–0.25 represents little if any, 0.26–0.49 weak, 0.50–0.69 moderate, 0.70–0.89 strong and 0.90–1.00 very strong correlation [27]. According to the COSMIN criteria, construct validity of the mPDQ-NL Shoulder is sufficient if 75% of predefined hypotheses are met [25,28].

#### Reliability

**Internal consistency.** The degree of the interrelatedness among the items of the mPDQ-NL Shoulder was determined by calculating the Cronbach’s Alpha for the overall score on the questionnaire and for the VAS pain score [13,29,30]. Good internal consistency is generally considered when Cronbach’s alpha lies between 0.70 and 0.95 [25].

**Measurement error.** Absolute measurement errors were determined by first calculating the standard error of measurement (SEM), which can be derived by dividing the standard deviation of the mean differences (SDdiff) between test and retest by $\sqrt{2}$ [31]. The smallest real change that can be detected in scores despite measurement error is represented by the smallest detectable change (SDC). The SDC with a 95% confidence interval (CI) for the individual level was calculated by $1.96\;x\;SEM\;x\;\sqrt{2}$, and at the group level by $1.96\;x\;SEM\;x\;\frac{\sqrt{2}}{\sqrt{n}}$ [32]. Absolute agreement was assessed using the Bland and Altman method [33] by calculating the mean difference between test and retest using the 95% CI interval, where zero lying within the 95% CI interval is considered as absolute agreement and zero lying outside the 95% CI interval indicates a systematic bias.

**Reliability.** Reliability was assessed using intraclass correlation coefficients (ICC) with the corresponding 95% confidence interval (CI) for the total score of the mPDQ-NL Shoulder. The ICC two-way random effects model, type agreement [34], was used. In general, an ICC value >0.70 indicates high reliability [24].

#### Floor and ceiling effects

Floor and ceiling effects were inspected. They are present when 15% of the participants achieve the minimum or maximum scores, respectively. This reduces the validity and reliability of an instrument, as participants with extreme scores cannot be distinguished from each other [25].

## Results

**Fig 1:**
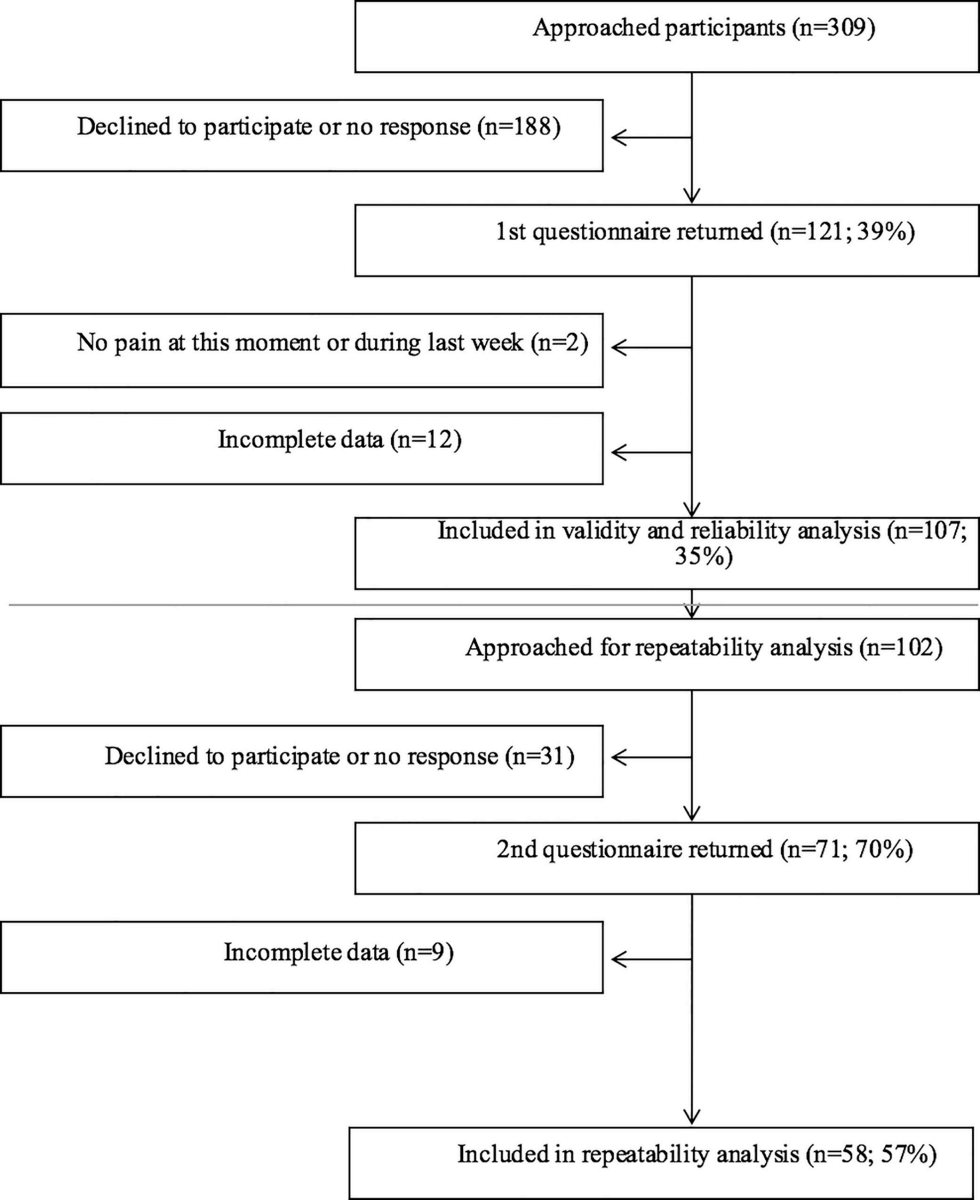
Flowchart showing patient inclusion.

In total, 309 patients were approached to participate in the study; 121 patients (39%) returned the questionnaires, 107 (35%) of whom were included in the validity and reliability analysis. For the reliability study 102 patients were approached. The response rate for the reliability analysis was 71 participants (70%), 58 (57%) of whom were included in the analysis. Two patients were excluded from the analysis, since an intervention was carried out between test-retest periods. At the first measurement, the number of cases lost because of missing items was 12 out of 121 respondents (10%). For the second assessment of the questionnaire this was 9 out of 71 respondents (13%). Flow chart for inclusion is shown in Fig 1.

Patient characteristics are presented in Table 2. Total scores of the questionnaires are presented in Table 3. A total of 20 (17%) patients show a likely neuropathic pain profile (mPDQ-NL Shoulder score ≥19) and 28 (24%) patients a possible neuropathic pain profile (mPDQ-NL Shoulder score 13 to 18). The median mPDQ-NL Shoulder score differed significantly between these profiles (p<0.001) (Table 3).

**Table 2:** Baseline characteristics.

	Included patients (n = 107)	Non-responders (n = 186)
Age (years)	53 ± 9 (24–77)	56 ± 12 (26–85)
Gender		
Male	48 (45%)	86 (46%)
Female	59 (55%)	100 (54%)
Body mass index (kg/m^2^)	27 ± 4 (17–44)	**
Duration of pain (months)*	24 (3–240)	**
Pain present in dominant arm		**
Left	9 (8.8%)	
Right	29 (28.4%)	

Mean ± SD (min-max) are shown for variables with normal distribution.

* Median (IQ range) is shown for variables with non-normal distribution. Gender and pain are shown as number of patients (%).

** No information known.

**Table 3:** Descriptive statistics questionnaires.

**Questionnaire**	**Total score on questionnaire**	**Min**	**Max**
mPDQ-NL Shoulder total score (n = 107)	13.3 ± 6.6	0	33
Score ≤ 12 (n = 58)	9.0 (IQR = 4.0) *		
Score 13–18 (n = 29)	16.0 (IQR = 3.0) *		
Score ≥ 19 (n = 20)	22.0 (IQR = 6.5) *		
S-LANSS total score(n = 102)	8.0 (IQR = 8.0) *	0	24
DASH total score (n = 98)	45.4 ± 21.3	4	88
VAS pain at rest (n = 107)	4.1 ± 2	0	10
VAS pain during physical activity (n = 107)	66.7 ± 2.1	1	10

Mean ± SD are shown for variables with normal distribution.

* Median and Interquartal range (IQR) are shown for variables with non-normal distribution.

### Construct validity

#### Structural validity

Three factors with an eigenvalue ≥1 were revealed with exploratory factor analysis. The first factor contained the items *burning sensation*, *tingling or prickling sensation*, *pain at light touch*, *sudden pain attacks*, *pain at cold or warm stimulus*, *numbness and pain at light pressure*. The second factor consisted of one item, *pain pattern*, and the third factor consisted of *pain radiation using a body map*. See Table 1 in S4 Appendix for the factor loadings and distribution among components.

#### Hypotheses testing

The correlations between the mPDQ-NL Shoulder and the S-LANSS, DASH, VAS pain at rest and VAS pain during physical activity are presented in Table 1, all correlations were significant (P<0.001). A moderate correlation (0.55) was seen between the mPDQ-NL Shoulder and the S-LANSS and VAS physical activity. Weak correlations (0.36–0.47) were seen between the mPDQ-NL Shoulder and the DASH and VAS pain at rest. Of all predefined hypotheses, 86% could be confirmed.

### Reliability

#### Internal consistency

Cronbach’s alphas were 0.8 for the overall mPDQ-NL Shoulder questionnaire, and 0.9 for the VAS pain score.

#### Measurement error and reliability

**Fig 2:**
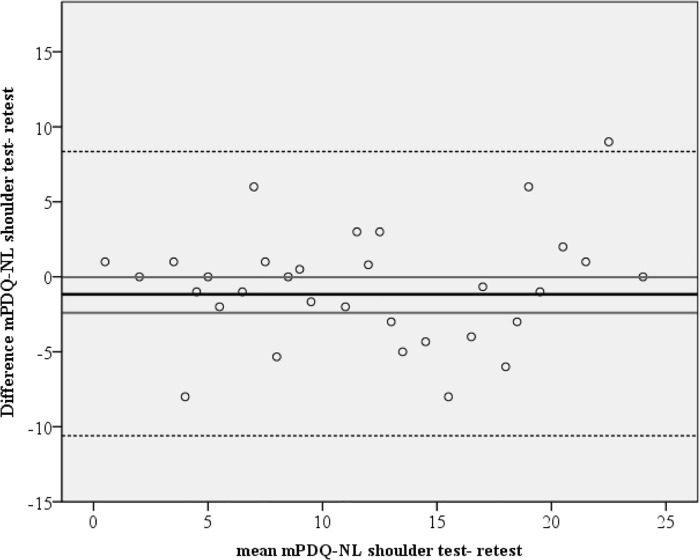
Bland Altman plot. Bland Altman plot with 95% confidence interval (CI). Vertical axis: difference between mPDQ-NL Shoulder test and retest. Horizontal axis: mean mPDQ-NL Shoulder when combining test and retest. The black horizontal line represents the mean difference between test and retest. The gray lines represent the 95% CI of this mean difference of the mPDQ-NL Shoulder. The dotted lines represent the limits of agreement.

Reliability measures for the mPDQ-NL Shoulder are presented in Table 4. For the total mPDQ-NL Shoulder score, SEM was 3.4, SDCind 9.5 and SDCgroup 1.2. The mean difference of the mPDQ-NL Shoulder total score between test and retest was -1.1 and the corresponding 95% CI did not contain zero, indicating systematic bias. The Bland Altman plot is presented in Fig 2. ICC value of the total score was 0.7.

**Table 4:** Measurement error and reliability of mPDQ-NL Shoulder (n = 58).

Baseline mean ± SD	13.3 ± 6.6
Retest mean ± SD	11.2 ± 5.9
Mean difference (95% CI)	-1.1 (-2.4; -0.03)
SEM	3.4
SDC_ind_	9.5
SDC_grp_	1.2
ICC (95% CI)	0.7 (0.5–0.8)

SD: standard deviation; CI: confidence interval; SEM: standard error of measurement; SDC_ind_: smallest detectable change at the individual level; SDC_grp_: smallest detectable change at the group level; ICC: intraclass correlation coefficient.

### Floor and ceiling effects

No floor (lowest score: 2%, n = 2) or ceiling effects (highest score: 0%, n = 0) were seen for the total mPDQ-NL Shoulder score. No ceiling effects were seen in any of the items.

### Subgroups

Analyses of the subgroups showed a significant difference based on scores of the mPDQ-NL Shoulder (X^2^ = 86.6; df = 2; p <0.001). Baseline characteristics and differences of the posthoc analyses of the subgroups are shown in Table 5. Gender and age showed no significant differences between the subgroups.

**Table 5:** Baseline characteristics and differences of the subgroups based on the mPDQ-NL Shoulder score.

**Subgroup mPDQ-NL Shoulder**	**≤12 points** **(n = 58)**	**13–18 points** **(n = 29)**	**≥ 19 points** **(n = 20)**	Kruskall Wallis***	**Posthoc** **≤12 vs 13–18#**	**Posthoc** **≤12 vs ≥19#**	**Posthoc** **13–18 vs ≥19#**
Age	55 ± 10	54 ± 9	51 ± 11	X^2^ = 2.4; df = 2; P = 0.30			
Gender				X^2^ = 0.2; df = 2; P = 0.90			
Male	27 (46.6%)	12 (41.4%)	9 (45.0%)				
Female	31 (53.4%)	17 (58.6%)	11 (55.0%)				
BMI	27 ± 5 (18–45)	26 ± 4 (18–33)	27 ± 6 (19–39)	X^2^ = 0.6; df = 2; P = 0.73			
SLANSS	5.4 (IQR = 7)*	9.4 (IQR = 6)*	13.5 (IQR = 12)*	X^2^ = 22.2; df = 2; P<0.001	U = 444.5; Z = -3.1; ***P = 0*.*002***	U = 191.5; Z = -4.3; ***P<0*.*001***	U = 197.5; Z = -1.7; P = 0.08
DASH	37.9 ± 19.6**	47.6 ± 20.8**	63.9 ± 14.5**	X^2^ = 19.3; df = 2; P<0.001	U = 532.5; Z = -1.9; P = 0.06	U = 152.5; Z = -4.3; ***P<0*.*001***	U = 132.5; Z = -2.6; P = 0.01
VAS pain at rest	3.4 ± 2.6**	3.6 ± 1.9**	6.8 ± 1.9**	X^2^ = 23.6; df = 2; P<0.001	U = 763; Z = -0.7; P = 0.5	U = 200; Z = -4.4; ***P<0*.*001***	U = 68; Z = -4.6; ***P<0*.*001***
VAS pain during activity	6.0 ± 2.2**	6.8 ± 1.5**	8.6 ± 1.1**	X^2^ = 26.0; df = 2; P<0.001	U = 691.5; Z = -1.4; P = 0.2	U = 161; Z = -4.9; ***P<0*.*001***	U = 98; Z = -4.0; ***P<0*.*001***

* Median and Interquartal range (IQR) are shown for variables with non-normal distribution

** Mean ± SD are shown for variables with normal distribution

*** X^2^ = Chi-square distribution

df = degrees of freedom

# U = Mann-Whitney U test

Z = z-score; *Bold/italic*: significant difference, p-value corrected for multiple comparisons, corrected p-value = P< 0.003.

## Discussion

This study is the first to provide a Dutch mPDQ to fit patients with SAPS with a first step toward individualized treatment, and to assess its construct validity and reliability. It shows to be a valid and fairly reliable patient-reported instrument for identifying patients with SAPS with a possible neuropathic pain component.

Overall validity of the mPDQ-NL Shoulder can be considered good, meaning that the questionnaire measures what it intends to. The findings of the present study regarding structural validity of the mPDQ-NL Shoulder are in line with the Japanese, Turkish and Spanish PDQs and the Dutch modified PDQ Hip and Knee, although some small differences were seen with respect to factor loadings [15,29,30,35]. The mPDQ-NL Shoulder exists of three factors compared to two in the original PDQ. However, the second and third factor—both existing of one item—make up the second factor in the original version. In this version Freyenhagen et al emphasize the relevance of the first factor [13].

Construct validity of the mPDQ-NL Shoulder was good, with 86% of the predefined hypotheses confirmed. The highest correlation was expected between mPDQ-NL Shoulder and the S-LANSS, as both measure neuropathic pain components [36], and also based on the results of a previous study by Hochman et al. [37]. Yet only a moderate correlation was found. This might be explained by some important differences between the two instruments, i.e. the Likert items in the joint-specific mPDQ-NL Shoulder with equal weighting system versus the binary items in the S-LANSS, used for any cause of chronic pain, with a weighted scoring system. However, the found correlation is in line with the Dutch modified PDQ Hip and Knee [38].

Regarding reliability, internal consistency, measurement error and reliability were assessed. Internal consistency was good (Cronbach’s alpha 0.8). Cronbach’s alpha of the original PDQ was not available for comparison.

With a range for the total mPDQ-NL Shoulder score from -1 to 38 points, a 3.4 SEM seems acceptable. An SDC value at the individual level of 9.5 and a low SDC value at the group level of 1.2 indicate that the mPDQ-NL Shoulder might be useful for group comparisons rather than detecting changes at the individual level. To distinguish clinically important changes from measurement errors, the SDC values should be smaller than the minimal important change (MIC) [25]. As the MIC of the painDETECT has not yet been determined, it was not possible to properly assess whether the SEM and SDC values are acceptable. Overall, the SEM and SDC values were comparable to the findings of Rienstra et al. for the mPDQ Hip and Knee [15]. Although the corresponding 95% CI of the mean difference of the mPDQ-NL Shoulder total score between the two measurements almost reached zero (-2.4 to -0.03), it did not contain zero, indicating a possible systematic measurement bias between test and retest. This bias might be related to the varying levels of pain intensity over time in patients with subacromial pain. In addition, reliability was good with an ICC of 0.7, although previous reliability studies of the Japanese and Spanish PDQ and the Dutch mPDQ-NL Hip and Knee show overall ICCs of 0.90–0.94 [15,29,30]. We did use a recommended time interval of two weeks, and in this rather small period no change in the complaints pattern was to be expected. However, as we did not use a global rating of change score to quantify improvement or deterioration in the patient’s health condition between test and retest, we cannot ensure that no actual change occurred. This might explain the systematic measurement bias and the lower ICC in comparison to other studies.

No significant floor and ceiling effects were observed for total mPDQ-NL Shoulder scores. The floor and ceiling effects of our study are comparable with those found for the mPDQ-NL Hip and Knee [15].

With the development of the mPDQ-NL Shoulder we can now identify patients with a neuropathic pain profile in SAPS. Patients with other chronic pain conditions might be more at risk to also develop a neuropathic pain syndrome of the shoulder. As we excluded them in the current study, this needs to be addressed in further research. A limitation of our study is the use of the S-LANSS in the validation study, which is validated for English but translated into Dutch according to the guidelines of Beaton for this purpose. Furthermore, the study has a high rate of non-responders, but demographics (age and gender) of these patients are comparable to those of the included patients. A reason for not responding might be having mailed the questionnaires without informing the patient in person at the outpatient clinic. Moreover, as we did not investigate responsiveness, whether the tool can record change over time with a sensitivity-to-change analysis has to further assessed. In addition, we did not use neurological, electrophysiological or psychophysical tests to demonstrate the presence of neuropathic pain in our population. Such tests have been used before in other validation studies. Though, these show that, for example, pain pressure thresholds seem to measure different aspects of the pain experience compared to patient-reported assessment of pain experience [38].

Identification of patients with a neuropathic pain profile has important implications, especially considering this population is perhaps larger than expected, with 41% of patients showing a possible or likely neuropathic pain profile in the current study. In addition, these patients with a neuropathic pain profile tended to experience more symptoms. The subgroup analyses showed a significant difference in reported pain between patients without or a possible neuropathic pain profile compared to patients with a likely neuropathic pain profile. Higher reported pain in patients could predict to patients with a neuropathic pain pattern in SAPS. As a result it may facilitate customized treatment, with different kinds of neuromodulating medication, and it may prevent unwanted surgery. In future research, it may assist in assessing the true effectiveness of treatment when stratification of patients with and without neuropathic pain-like symptoms is possible.

## Conclusion

The mPDQ-NL Knee was adapted to fit Dutch patients with SAPS. It shows good construct validity, internal consistency and reliability. However, a systemic bias might be present. To conclude, this study showed that the mPDQ-NL Shoulder seems to reflect neuropathic-like pain symptoms experienced by patients with SAPS. Whether it also may be used as a measure to record change over time or after treatment has to be further assessed.

## Supporting information

S1 Appendix mPDQ-NL Shoulder left. (PDF)

S2 Appendix mPQD-NL shoulder right. (PDF)

S3 Appendix mPDQ-NL shoulder left and right (translated to English). (PDF)

S4 Appendix Factor loadings and distribution among components. (PDF)

S1 Database (SAV)

## References

[1] Van der Windt DAWM, Koes BW, de Jong BA, Bouter LM. Shoulder disorders in general practice: incidence, patient characteristics, and management. Ann Rheum Dis 1995;54:959–64 8546527 10.1136/ard.54.12.959 PMC1010060

[2] Virta L, Joranger P, Brox JI, Eriksson R. Costs of shoulder pain and resource use in primary health care: a cost-of-illness study in Sweden. BMC Musculoskelet Disord. 2012 2 10;13:17 10.1186/1471-2474-13-17 22325050 PMC3299609

[3] Bigiliani LU, Levine WN. Subacromial impingement syndrome. J Bone Joint Surg Am 1997;79:1854–68 9409800

[4] Cummins CA, Sasso LM, Nicholson D. Impingement syndrome: temporal outcomes of nonoperative treatment. J Shoulder Elbow Surg. 2009 Mar-Apr;18(2):172–7. 10.1016/j.jse.2008.09.005 19095464

[5] Kuijpers T, van der Windt DA, Boeke AJ, Twisk JW, Vergouwe Y, Bouter LM et al Clinical prediction rules for the prognosis of shoulder pain in general practice. Pain. 2006 2;120(3):276–85. 10.1016/j.pain.2005.11.004 16426760

[6] Diercks RL, Bron C, Dorrestijn O, Meskers C, Naber R, de Ruijter T et al Guideline for diagnosis and treatment of subacromial pain syndrome: a multidisciplinary review by the Dutch Orthopaedic Association. Acta Orthop 2014 6;85(3):314–22 10.3109/17453674.2014.920991 24847788 PMC4062801

[7] Dorrestijn O, Stevens M, Winters JC, van der Meer K, Diercks RL. Conservative or surgical treatment for subacromial impingement syndrome? A systematic review. J Shoulder Elbow Surg 2009;18:652–60. 10.1016/j.jse.2009.01.010 19286397

[8] Ketola S, Lehtinen J, Arnala I, Nissinen M, Westenius H, Sintonen H et al Does arthroscopic acromioplasty provide any additional value in the treatment of shoulder impingement syndrome? A two year randomized controlled trial. J Bone Joint Surg Br 2009;91:1326–34. 10.1302/0301-620X.91B10.22094 19794168

[9] Curatolo M, Arendt-Nielsen L, Petersen-Felix S. Central hypersensitivity in chronic pain: mechanisms and clinical implications. Phys Med Rehabil Clin North Am 2006;17:287–302. 10.1016/j.pmr.2005.12.010 16616268

[10] Gwilym SE, Oag HC, Tracey I, Carr AJ. Evidence that central sensitisation is present in patients with shoulder impingement syndrome and influences the outcome after surgery. J Bone Joint Surg Br. 2011 4;93(4):498–502. 10.1302/0301-620X.93B4.25054 21464489

[11] Latremoliere A, Woolf CJ. Central sensitization: a generator of pain hypersensitivity by central neural plasticity. J Pain. 2009;10:895–926. 10.1016/j.jpain.2009.06.012 19712899 PMC2750819

[12] Paul TM, Soo Hoo J, Chae J, Wilson RD. Central hypersensitivity in patients with subacromial impingement syndrome. Arch Phys Med Rehabil. 2012 12;93(12):2206–9. 10.1016/j.apmr.2012.06.026 22789774 PMC3508388

[13] Freynhagen R, Baron R, Gockel U, Tölle TR. Pain DETECT: a new screening questionnaire to identify neuropathic components in patients with back pain. Current Medical Research and Opinion. 2006; 22: 1911–1920. 10.1185/030079906X132488 17022849

[14] Hochman JR, Davis AM, Elkayam J, Gagliese L, Hawker GA. Neuropathic pain symptoms on the modified painDETECT correlate with signs of central sensitization in knee osteoarthritis. Osteoarthritis Cartilage. 2013 9;21(9):1236–42. 10.1016/j.joca.2013.06.023 23973136

[15] Rienstra W, Blikman T, Mensink FB, van Raay JJ, Dijkstra B, Bulstra SK, et al The Modified painDETECT Questionnaire for Patients with Hip or Knee Osteoarthritis: Translation into Dutch, Cross-Cultural Adaptation and Reliability Assessment. PLoS ONE. 2015;10(12):e0146117 10.1371/journal.pone.0146117 26720417 PMC4697818

[16] Bennett M. The LANSS Pain Scale: the Leeds assessment of neuropathic symptoms and signs. Pain. 2001 5;92(1–2):147–57. 11323136 10.1016/s0304-3959(00)00482-6

[17] Bennett MI, Smith BH, Torrance N, Potter J. The S-LANSS score for identifying pain of predominantly neuropathic origin: validation for use in clinical and postal research. The Journal of Pain. 2005;6(3):149–58. 10.1016/j.jpain.2004.11.007 15772908

[18] Hudak PL, Amadio PC, Bombardier C. Development of an upper extremity outcome measure: the DASH (disabilities of the arm, shoulder and hand) [corrected]. The Upper Extremity Collaborative Group (UECG) Am J Ind Med. 1996 6;29(6):602–8. Erratum in: Am J Ind Med 1996 Sep;30(3):372. 10.1002/(SICI)1097-0274(199606)29:6<602::AID-AJIM4>3.0.CO;2-L 8773720

[19] Palmen CM, Meijden E van der, Nelissen Y, Koke AJA. De betrouwbaarheid en validiteit van de Nederlandse vertaling van de Disability of the Arm, Shoulder and Hand questionniare (DASH). Ned Tijdschr Fysiother, 2004, 114:30–35.

[20] Price DD, McGrath PA, Rafii A, Buckingham B. The validation of visual analogue scales as ratio scale measures for chronic and experimental pain. Pain. 1983;17(1):45–56. 6226917 10.1016/0304-3959(83)90126-4

[21] Jones RC 3rd, Backonja MM. Review of neuropathic pain screening and assessment tools. Curr Pain Headache Rep. 2013 9;17(9):363,013-0363-6. 10.1007/s11916-013-0363-6 23996692

[22] Mulvey MR, Bennett MI, Liwowsky I, Freynhagen R. The role of screening tools in diagnosing neuropathic pain. Pain Manag. 2014 5;4(3):233–43. 10.2217/pmt.14.8 24953075

[23] Beaton DE, Bombardier C, Guillemin F, Ferraz MB. Guidelines for the process of cross cultural adaptation of self-report measures. Spine. 2000;25(24):3186–91. 11124735 10.1097/00007632-200012150-00014

[24] Terwee CB, Mokkink LB, Knol DL, Ostelo RW, Bouter LM, de Vet HC. Rating the methodological quality in systematic reviews of studies on measurement properties: a scoring system for the COSMIN checklist. Qual Life Res. 2012; 21: 651–657. 10.1007/s11136-011-9960-1 21732199 PMC3323819

[25] Terwee CB, Bot SD, de Boer MR, van der Windt DA, Knol DL, Dekker J, et al Quality criteria were proposed for measurement properties of health status questionnaires. J Clin Epidemiol. 2007; 60: 34–42. 10.1016/j.jclinepi.2006.03.012 17161752

[26] Beezer RA. Eigenvalues and Eigenvectors. In: A first course in linear algebra, version 3.50 (Created: 2015-12-30T15:06:58–08:00; Technical Refresh: 2017-04-14) Free online book under GNU licence, University of Puget Sound.

[27] Domholdt E. Physical therapy research, principles and applications. 2nd ed Philadelphia: WB Saunders; 2000.

[28] de Vet HC, Terwee CB, Mokkink LB, Knol DL. Reviews of measurement properties In: Measurement in medicine: a practical guide. Cambridge University Press; 2011: 300. ISBN: 0521133858

[29] De Andrés J, Pérez-Cajaraville J, Lopez-Alarcón MD, López-Millán JM, Margarit C, Rodrigo-Royo MD et al Cultural adaptation and validation of the painDETECT scale into Spanish. Clin J Pain. 2012 Mar-Apr; 28: 243–53. 10.1097/AJP.0b013e31822bb35b 21926908

[30] Matsubayashi Y, Takeshita K, Sumitani M, Oshima Y, Tonosu J, Kato S et al Validity and reliability of the Japanese version of the painDETECT questionnaire: a multicenter observational study. PLoS One. 2013; 8 10.1371/journal.pone.0068013 24098629 PMC3787034

[31] de Vet HC, Terwee CB, Knol DL, Bouter LM. When to use agreement versus reliability measures. J Clin Epidemiol. 2006–10; 59: 1033–9. 10.1016/j.jclinepi.2005.10.015 16980142

[32] de Vet HC, Bouter LM, Bezemer PD, Beurskens AJ. Reproducibility and responsiveness of evaluative outcome measures. theoretical considerations illustrated by an empirical example. Int J Technol Assess Health Care. 2001; 17: 479–487. 11758292

[33] Bland J M JM, Bland JM, Altman DG. Statistical methods for assessing agreement between two methods of clinical measurement. Lancet. 1986-2-8; 1: 307–10. 2868172

[34] Mcgraw K, Wong SP. Forming inferences about some intraclass correlation coefficients. Psychol Methods. 1996; 1: 30–46.

[35] Alkan H, Ardic F, Erdogan C, Sahin F, Sarsan A, Findikoglu G. Turkish version of the painDETECT questionnaire in the assessment of neuropathic pain: a validity and reliability study. Pain Med. 2013;14: 1933–1943. 10.1111/pme.12222 23924395

[36] Campbell DT, Fiske DW. Convergent and discriminant validation by the multitrait-multimethod matrix. Psychol Bull. 1959; 56: 81–105. 13634291

[37] Hochman J, Gagliese L, Davis A, Hawker G. Neuropathic pain symptoms in a community knee OA cohort. Osteoarthritis and Cartilage. 2011;19(6):647–54. 10.1016/j.joca.2011.03.007 21440077

[38] Rienstra W, Blikman T, Dijkstra B, van Raay J, Slager G, Bulstra S et al Validity of the Dutch modified painDETECT questionnaire for patients with hip or knee osteoarthritis. Disabil Rehabil. 2017; 8:1–7. 10.1080/09638288.2017.1413429 29221427

