# Supplementary material for: Adaptation, validity and reliability of the modified painDETECT questionnaire for patients with subacromial pain syndrome
Source: PLoS One. 2019 Feb 6;14(2):e0211880. doi: 10.1371/journal.pone.0211880 (PMC6364996; doi:10.1371/journal.pone.0211880)
Supplement: S1 Appendix — (PDF) [file pone.0211880.s001.pdf]

Om inzicht te krijgen in de aard van uw pijnklachten, willen wij u vragen om de onderstaande vragenlijst in te vullen. Wanneer u de vragenlijst volledig hebt ingevuld, kunt u deze inleveren bij de balie.

Vult u alstublieft de datum van vandaag in

STICKER

Datum:_____________________

Modified painDETECT LINKER SCHOUDER

SCORE:

We hebben gemerkt dat mensen verschillende woorden gebruiken om verschillende soorten van pijn bij schouderklachten te beschrijven.
Naast pijn beschrijven sommige mensen vervelende klachten zoals schrijnende pijn, stijfheid en zelfs tintelingen rondom hun gewrichten. Vanaf nu, gebruiken we simpelweg het woord "pijn" voor *alle* vervelende klachten.

Deze vragenlijst vraagt naar symptomen van kapselproblemen van de schouder. We zullen hier vragen naar symptomen in rust, niet naar uw symptomen tijdens lichamelijke activiteit.
De gegeven beschrijvingen kunnen, maar hoeven niet overeen te komen met uw "pijn", ongeacht hoe ernstig deze is. Laat alstublieft geen enkele vraag onbeantwoord, zodat we zeker kunnen zijn over uw symptomen.

Denkt u alstublieft ALLEEN aan uw LINKER SCHOUDER wanneer u de volgende vragen beantwoordt.

1. Straalde de "pijn" van uw LINKER schouder naar boven of naar beneden uit richting uw nek, rug of naar uw LINKER arm in de AFGELOPEN WEEK?
(Vink (√) hieronder JA of NEE aan) **LINKER schouder**

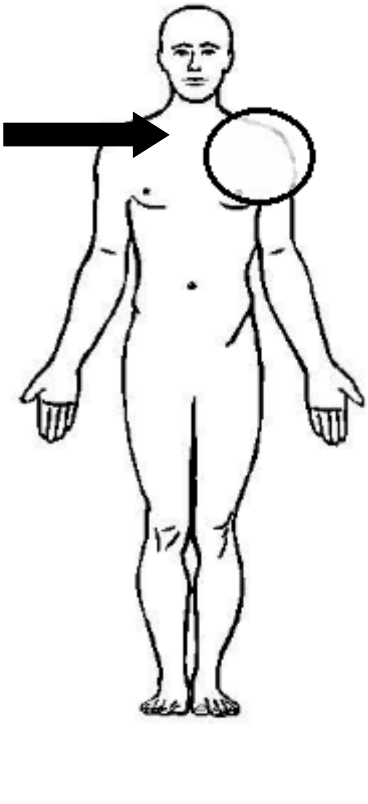

JA [+2] → JA Zo " JA ", markeer alstublieft de "pijnlijke" gebieden van uw nek, rug of LINKER arm op het hiernaast staande plaatje

NEE [0] → NEE Als u "NEE" heeft aangevinkt, ga dan op de volgende bladzijde verder met Vraag 2.

2. Kruis één afbeelding aan die het verloop van de “pijn” aan uw LINKER schouder in de AFGELOPEN WEEK het beste weergeeft: (Vink (√) één hokje aan die het beste bij u pijn past)

[0] 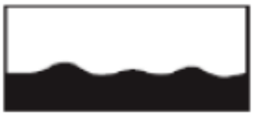 Aanhoudende pijn met lichte schommelingen

[-1] 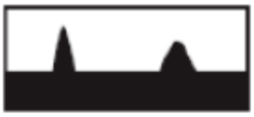 Aanhoudende pijn met pijnaanvallen

[+1] 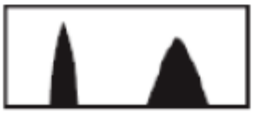 Pijnaanvallen, daartussen pijnvrij

[+1] 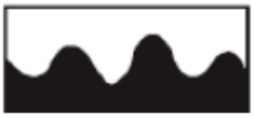 Pijnaanvallen, daartussen pijn

3. Kies alstublieft het antwoord dat het beste de aard van de “pijn” aan uw LINKER schouder in de AFGELOPEN WEEK beschrijft.

Denk eraan dat we het woord ‘pijn’ gebruiken voor alle vervelende klachten die u zou kunnen beschrijven als pijn. **Laat u alstublieft geen vragen onbeantwoord.**
(Vink (√) één hokje aan die het beste bij u klacht past)

a. Hebt u last van een branderig gevoel (bijvoorbeeld als van brandnetels) in of rondom uw LINKER schouder?

Nooit [0] Vrijwel niet [+1] Licht [+2] Matig [+3] Sterk [+4] Zeer sterk [+5]

b. Hebt u een kriebelend of prikkelend gevoel in het pijnlijke gebied van uw LINKER schouder (zoals mieren, zwakke stroom)?

Nooit ☐ Vrijwel niet ☐ Licht ☐ Matig ☐ Sterk ☐ Zeer sterk ☐

c. Is een lichte aanraking (kleding, beddengoed) in dit gebied pijnlijk?

Nooit ☐ Vrijwel niet ☐ Licht ☐ Matig ☐ Sterk ☐ Zeer sterk ☐

d. Hebt u schietende pijnaanvallen in het pijnlijke gebied van uw LINKER schouder, als elektrische scheuten?

Nooit ☐ Vrijwel niet ☐ Licht ☐ Matig ☐ Sterk ☐ Zeer sterk ☐

e. Is kou of warmte (badwater) in dit gebied wel eens pijnlijk?

Nooit ☐ Vrijwel niet ☐ Licht ☐ Matig ☐ Sterk ☐ Zeer sterk ☐

d. Hebt u last van een doof gevoel in of rondom uw LINKER schouder?

Nooit ☐ Vrijwel niet ☐ Licht ☐ Matig ☐ Sterk ☐ Zeer sterk ☐

e. Wekt een lichte druk, bijvoorbeeld met de vinger, in dit gebied pijn op?

Nooit ☐ Vrijwel niet ☐ Licht ☐ Matig ☐ Sterk ☐ Zeer sterk ☐

4. In de volgende paar vragen kunt u ons vertellen over de mate van ongemak die u ervaart in of rondom uw LINKER schouder op een 0-10 schaal. 0 staat voor 'geen pijn' en 10 is 'onuitstaanbare, nauwelijks verdraagbare pijn'
Omcirkel bij elke vraag slechts EEN cijfer.

a. Welk cijfer geeft u op dit moment de pijn aan uw LINKER schouder .

Geen 'pijn' **Onuitstaanbare 'pijn'**

0	1	2	3	4	5	6	7	8	9	10

b. Hoe intens was de HEFTIGSTE 'pijn' in de AFGELOPEN WEEK aan uw LINKER schouder.

Geen 'pijn' **Onuitstaanbare 'pijn'**

0	1	2	3	4	5	6	7	8	9	10

c. Hoe intens was GEMIDDELD de 'pijn' in de AFGELOPEN WEEK aan uw LINKER schouder.

Geen 'pijn' **Onuitstaanbare 'pijn'**

0	1	2	3	4	5	6	7	8	9	10

Dit is het einde van de vragenlijst. Controleert u alstublieft of alle vragen zijn beantwoord.

Hartelijk dank voor het invullen.
