# Supplementary material for: Adaptation, validity and reliability of the modified painDETECT questionnaire for patients with subacromial pain syndrome
Source: PLoS One. 2019 Feb 6;14(2):e0211880. doi: 10.1371/journal.pone.0211880 (PMC6364996; doi:10.1371/journal.pone.0211880)
Supplement: S3 Appendix — (PDF) [file pone.0211880.s003.pdf]

To gain insight into the nature of your pain, we would like to ask you to complete the questionnaire below. When you have completed the questionnaire completely, you can hand it in at the desk.

Please enter today's date

STICKER

Date:______________________

Modified painDETECT LEFT SHOULDER

SCORE:

We have noticed that people use different words to describe different types of pain associated with shoulder complaints.
In addition to pain, some people describe symptoms such as stiffness, burning or even tingling. From now on, we use simply the word 'pain' for all annoying complaints you can experience.

This questionnaire asks for symptoms of your shoulder. We will ask for symptoms at rest, not for symptoms experienced during physical activity.
The descriptions giving can, but do not have to correspondent with your pain, regardless of how severe it is. Please do not leave any questions unanswered, so we can be sure about your symptoms.

Please think of your left shoulder only when answering the following questions.

1. Does your pain of the left shoulder radiate to others regions (e.g., neck, back or arm?) of your body during the past week? (Check (√) below YES or NO)

LEFT shoulder

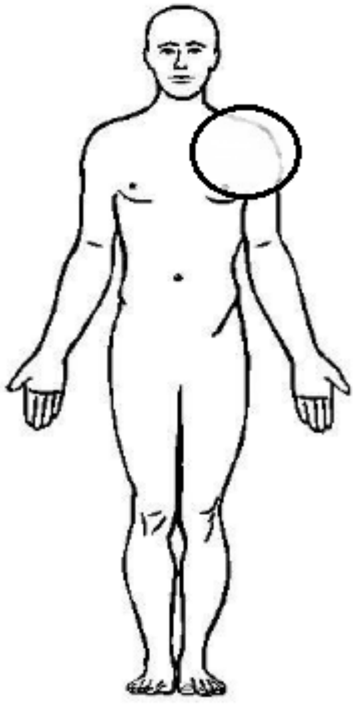

YES ☐ → If yes, please draw the direction in which the pain radiates on the picture

NO ☐ → If no, please move on to question number 2 on the next page

2. Mark the picture that best describes the course of your pain of the left shoulder during the past week. (Please check (√) one box that most suits your complaints)

☐ 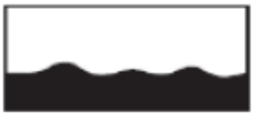 Persistent pain with slight fluctuations

☐ 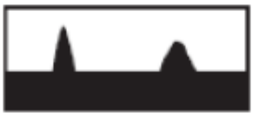 Persistent pain with pain attacks

☐ 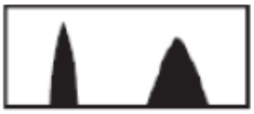 Pain attacks without pain between them

☐ 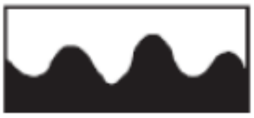 Pain attacks with pain between them

3. Please choose the answer that best describes the nature of the pain of your left shoulder in the past week.

Remember that we use the word 'pain' for all the nasty complaints you can experience as pain. Please do not leave any questions unanswered.
(Please check (√) one box per question that most suits your complaints)

a. Do you suffer from a burning sensation (e.g. stinging nettles) in the marked area of your left shoulder?

never ☐ hardly noticed ☐ slightly ☐ moderately ☐ strongly ☐ very strongly ☐

b. Do you have a tingling or prickling sensation in the area of left shoulder (like crawling ants or electrical tingling)?

never ☐ hardly noticed ☐ slightly ☐ moderately ☐ strongly ☐ very strongly ☐

c. Is light touching (clothing, a blanket) in this area of your left shoulder painful?

never ☐ hardly noticed ☐ slightly ☐ moderately ☐ strongly ☐ very strongly ☐

d. Do you have sudden pain attacks in the area of your left shoulder, like electric shocks?

never ☐ hardly noticed ☐ slightly ☐ moderately ☐ strongly ☐ very strongly ☐

e. Is cold or heat (bath water) in the area of your left shoulder occasionally painful?

never ☐ hardly noticed ☐ slightly ☐ moderately ☐ strongly ☐ very strongly ☐

d. Do you suffer from a sensation of numbness in the areas that you marked?

never ☐ hardly noticed ☐ slightly ☐ moderately ☐ strongly ☐ very strongly ☐

e. Does slight pressure in this area of your left shoulder, e.g., with a finger, trigger pain?

never ☐ hardly noticed ☐ slightly ☐ moderately ☐ strongly ☐ very strongly ☐

4. The next questions ask about the level of discomfort you experience in or around you left shoulder on a 0-10 scale, where 0 stands for ‘no pain’ and 10 for ‘barely tolerable pain’.
<u>Please circle only one digit for each question.</u>

a. How would you assess your pain of the left shoulder now, at this moment?

None **Max.**

0	1	2	3	4	5	6	7	8	9	10

b. How strong was the strongest pain of the left shoulder during the past 4 weeks?

None **Max.**

0	1	2	3	4	5	6	7	8	9	10

c. How strong was the pain of the left shoulder during the past 4 week on average?

None **Max.**

0	1	2	3	4	5	6	7	8	9	10

This is the end of the questionnaire. Please check that all questions have been answered.

Thank you for filling out the form.

To gain insight into the nature of your pain, we would like to ask you to complete the questionnaire below. When you have completed the questionnaire completely, you can hand it in at the desk.

Please enter today's date

STICKER

Date:______________________

Modified painDETECT RIGHT SHOULDER

SCORE:

We have noticed that people use different words to describe different types of pain associated with shoulder complaints.
In addition to pain, some people describe symptoms such as stiffness, burning or even tingling. From now on, we use simply the word 'pain' for all annoying complaints you can experience.

This questionnaire asks for symptoms of your shoulder. We will ask for symptoms at rest, not for symptoms experienced during physical activity.
The descriptions giving can, but do not have to correspondent with your pain, regardless of how severe it is. Please do not leave any questions unanswered, so we can be sure about your symptoms.

Please think of your right shoulder only when answering the following questions.

1. Does your pain of the right shoulder radiate to others regions (e.g., neck, back or arm?) of your body during the past week? (Check (√) below YES or NO)

RIGHT shoulder

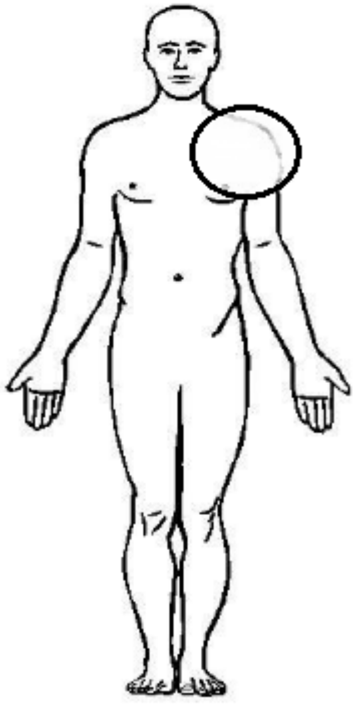

YES ☐ → If yes, please draw the direction in which the pain radiates on the picture

NO ☐ → If no, please move on to question number 2 on the next page

2. Mark the picture that best describes the course of your pain of the right shoulder during the past week. (Please check (√) one box that most suits your complaints)

☐ 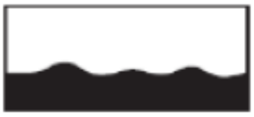 Persistent pain with slight fluctuations

☐ 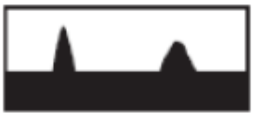 Persistent pain with pain attacks

☐ 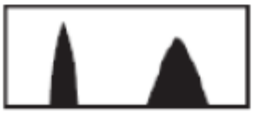 Pain attacks without pain between them

☐ 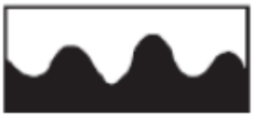 Pain attacks with pain between them

3. Please choose the answer that best describes the nature of the pain of your right shoulder in the past week.

Remember that we use the word 'pain' for all the nasty complaints you can experience as pain. Please do not leave any questions unanswered.
(Please check (√) one box per question that most suits your complaints)

a. Do you suffer from a burning sensation (e.g. stinging nettles) in the marked area of your right shoulder?

never ☐ hardly noticed ☐ slightly ☐ moderately ☐ strongly ☐ very strongly ☐

b. Do you have a tingling or prickling sensation in the area of right shoulder (like crawling ants or electrical tingling)?

never ☐ hardly noticed ☐ slightly ☐ moderately ☐ strongly ☐ very strongly ☐

c. Is light touching (clothing, a blanket) in this area of your right shoulder painful?

never ☐ hardly noticed ☐ slightly ☐ moderately ☐ strongly ☐ very strongly ☐

d. Do you have sudden pain attacks in the area of your right shoulder, like electric shocks?

never ☐ hardly noticed ☐ slightly ☐ moderately ☐ strongly ☐ very strongly ☐

e. Is cold or heat (bath water) in the area of your right shoulder occasionally painful?

never ☐ hardly noticed ☐ slightly ☐ moderately ☐ strongly ☐ very strongly ☐

d. Do you suffer from a sensation of numbness in the areas that you marked?

never ☐ hardly noticed ☐ slightly ☐ moderately ☐ strongly ☐ very strongly ☐

e. Does slight pressure in this area of your right shoulder, e.g., with a finger, trigger pain?

never ☐ hardly noticed ☐ slightly ☐ moderately ☐ strongly ☐ very strongly ☐

4. The next questions ask about the level of discomfort you experience in or around you right shoulder on a 0-10 scale, where 0 stands for 'no pain' and 10 for 'barely tolerable pain'.
Please circle only one digit for each question.

a. How would you assess your pain of the right shoulder now, at this moment?

None **Max.**

0	1	2	3	4	5	6	7	8	9	10

b. How strong was the strongest pain of the right shoulder during the past 4 weeks?

None **Max.**

0	1	2	3	4	5	6	7	8	9	10

c. How strong was the pain of the right shoulder during the past 4 week on average?

None **Max.**

0	1	2	3	4	5	6	7	8	9	10

This is the end of the questionnaire. Please check that all questions have been answered.

Thank you for filling out the form.
