# Supplementary material for: Adaptation, validity and reliability of the modified painDETECT questionnaire for patients with subacromial pain syndrome
Source: PLoS One. 2019 Feb 6;14(2):e0211880. doi: 10.1371/journal.pone.0211880 (PMC6364996; doi:10.1371/journal.pone.0211880)
Supplement: S4 Appendix — (PDF) [file pone.0211880.s004.pdf]

Table 1. Results from the factor analysis with factor loadings.

	Component		
	1	2	3
**Pain pattern**	.202	.048	**.884**
**Pain radiation using a body map**	.019	**-.918**	.030
**Burning sensation**	**.735**	.151	.109
**Tingling or prickling sensation**	**.749**	.373	.001
**Pain at light touching**	**.719**	-.132	.002
**Sudden pain attacks**	**.560**	.090	-.478
**Pain at cold/heat**	**.760**	.001	-.012
**Numbness sensation**	**.689**	.087	.163
**Pain at light pressure**	**.435**	.378	.152

The bold numbers show the different components.
